# Decoding HiPSC-CM’s Response to SARS-CoV-2: mapping the molecular landscape of cardiac injury

**DOI:** 10.1186/s12864-024-10194-5

**Published:** 2024-03-12

**Authors:** Sicheng Chen, Zhenquan Fu, Kaitong Chen, Xinyao Zheng, Zhenyang Fu

**Affiliations:** 1 Department of Cardiology, Shantou Central Hospital, Shantou, 515031 China; 2 Department of Cardiology, The First Affiliated Hospital of Guangdong Pharmaceutical University, Guangzhou, 510080 China; 3 School of Information Engineering, Guangdong University of Technology, Guangzhou, 510006 China; 4 Shantou University Medical College, Shantou, 515041 China; 5 Department of Cardiology, Guangdong Provincial People’s Hospital (Guangdong Academy of Medical Sciences), Southern Medical University, Guangzhou, 510080 China; 6 Department of Cardiology, The Third Affiliated Hospital of Southern Medical University, Guangzhou, 510630 China

**Keywords:** SARS-CoV-2, hiPSC-CMs, Transcriptome analysis, Molecular mechanisms, Biological significance, Mitochondrial dysfunction

## Abstract

**Background:**

Acute cardiac injury caused by coronavirus disease 2019 (COVID-19) increases mortality. Acute cardiac injury caused by COVID-19 requires understanding how severe acute respiratory syndrome coronavirus 2 (SARS-CoV-2) directly infects cardiomyocytes. This study provides a solid foundation for related studies by using a model of SARS-CoV-2 infection in human induced pluripotent stem cell-derived cardiomyocytes (hiPSC-CMs) at the transcriptome level, highlighting the relevance of this study to related studies. SARS-CoV-2 infection in hiPSC-CMs has previously been studied by bioinformatics without presenting the full molecular biological process. We present a unique bioinformatics view of the complete molecular biological process of SARS-CoV-2 infection in hiPSC-CMs.

**Methods:**

To validate the RNA-seq datasets, we used GSE184715 and GSE150392 for the analytical studies, GSE193722 for validation at the cellular level, and GSE169241 for validation in heart tissue samples. GeneCards and MsigDB databases were used to find genes associated with the phenotype. In addition to differential expression analysis and principal component analysis (PCA), we also performed protein-protein interaction (PPI) analysis, functional enrichment analysis, hub gene analysis, upstream transcription factor prediction, and drug prediction.

**Results:**

Differentially expressed genes (DEGs) were classified into four categories: cardiomyocyte cytoskeletal protein inhibition, proto-oncogene activation and inflammation, mitochondrial dysfunction, and intracellular cytoplasmic physiological function. Each of the hub genes showed good diagnostic prediction, which was well validated in other datasets. Inhibited biological functions included cardiomyocyte cytoskeletal proteins, adenosine triphosphate (ATP) synthesis and electron transport chain (ETC), glucose metabolism, amino acid metabolism, fatty acid metabolism, pyruvate metabolism, citric acid cycle, nucleic acid metabolism, replication, transcription, translation, ubiquitination, autophagy, and cellular transport. Proto-oncogenes, inflammation, nuclear factor-kappaB (NF-κB) pathways, and interferon signaling were activated, as well as inflammatory factors. Viral infection activates multiple pathways, including the interferon pathway, proto-oncogenes and mitochondrial oxidative stress, while inhibiting cardiomyocyte backbone proteins and energy metabolism. Infection limits intracellular synthesis and metabolism, as well as the raw materials for mitochondrial energy synthesis. Mitochondrial dysfunction and energy abnormalities are ultimately caused by proto-oncogene activation and SARS-CoV-2 infection. Activation of the interferon pathway, proto-oncogene up-regulation, and mitochondrial oxidative stress cause the inflammatory response and lead to diminished cardiomyocyte contraction. Replication, transcription, translation, ubiquitination, autophagy, and cellular transport are among the functions that decline physiologically.

**Conclusion:**

SARS-CoV-2 infection in hiPSC-CMs is fundamentally mediated via mitochondrial dysfunction. Therapeutic interventions targeting mitochondrial dysfunction may alleviate the cardiovascular complications associated with SARS-CoV-2 infection.

**Supplementary Information:**

The online version contains supplementary material available at 10.1186/s12864-024-10194-5.

## Introduction

An infection caused by severe acute respiratory syndrome coronavirus 2 (SARS-CoV-2), coronavirus disease 2019 (COVID-19), triggers systemic inflammation throughout the body. In spite of the World Health Organization's declaration that COVID-19 is no longer a global health emergency, its high infectivity and mortality rates pose a significant public health threat. An acute cardiac injury caused by COVID-19 can cause acute heart failure, hemodynamic disturbances, and malignant arrhythmias [1]. A considerable amount of research has shown that SARS-CoV-2 directly infects and replicates within cardiomyocytes, resulting in injuries to them [1, 2]. A comprehensive understanding of the mechanisms of SARS-CoV-2 infection in cardiomyocytes is crucial to developing treatments to address acute cardiac injury caused by COVID-19. Therefore, investigating the mechanisms of direct viral infection in cardiomyocytes holds paramount importance.

SARS-CoV-2 infected human induced pluripotent stem cell-derived cardiomyocytes (hiPSC-CMs) are a promising model for replicating the virus's effects on cardiomyocytes [3]. Myocardial tissues directly obtained from patients have technical and ethical barriers, whereas animal tissues have reliability issues, however, hiPSC-CMs derived from human cells revealed more accurate effects of SARS-CoV-2 on human cardiomyocytes [3, 4]. Experimental tools have limitations because they focus on specific aspects, and individual studies do not provide a comprehensive understanding of SARS-CoV-2's effects on hiPSC-CMs. Employing high-throughput sequencing and transcriptome analysis to investigate SARS-CoV-2 infection in hiPSC-CMs enables the identification and quantification of gene expression variations, unveiling gene regulatory mechanisms. By utilizing this approach, an in-depth understanding of molecular mechanisms, regulatory pathways, and target genes involved in SARS-CoV-2 infection in hiPSC-CMs can be gained, shedding light on possible biological responses to SARS-CoV-2, such as molecular mechanisms of injury, metabolism, and regulation. As a result, combining transcriptome analysis methods with high-throughput sequencing can provide insights into complex interactions of viral and hiPSC-CMs. With a model of SARS-CoV-2 infection in hiPSC-CMs, we uncovered the mechanism of biological damage to the heart by COVID-19, providing a solid base for related research [3, 5].

The study by Navaratnarajah et al. (2021) also compared the GSE184715 and GSE150392 datasets and found that SARS-CoV-2 infection of hiPSC-CMs resulted in a significant down-regulation of the expression of cardiomyocyte skeletal proteins [5]. In previous bioinformatics studies, the results of the analyses have been presented mechanistically without addressing the whole molecular biological process [6–8]. The novelty of this study is to appropriately portray the complete molecular biological process of SARS-CoV-2 infection of hiPSC-CMs at the bioinformatics level. Due to this, critical scientific inquiries have gone unanswered. The pertinent data were analyzed in further depth to address this deficiency and comprehensively examine the biological significance of SARS-CoV-2 infection in hiPSC-CMs.

The purpose of this study is to uncover the complex biological functions, changes in signaling pathways, key molecular mechanisms, hub genes, and potential intervention targets associated with SARS-CoV-2 infected hiPSC-CMs.

## Materials and methods

### Data acquisition

RNA-seq datasets of interest: Gene Expression Omnibus (GEO) database was searched with the keyword “SARS-CoV-2 AND hiPSC-CMs” to find datasets of direct and simulated SARS-CoV-2 infection in hiPSC-CMs, with an expression profiling study type by high throughput sequencing. We finally selected GSE184715 and GSE150392 [5, 9].

RNA-seq datasets for validation: Searching the GEO database with the keyword “(SARS-CoV-2 OR COVID-19) AND (hiPSC-CMs OR cardiomyocytes OR hearts)”, we found datasets of direct and simulated SARS-CoV-2 infection in hiPSC-CMs or heart tissues, with the sequencing type of expression profiling by high throughput sequencing and the species of human. Finally, we selected GSE193722 and GSE169241 to validate hub genes at the cellular level and in COVID-19 heart samples, respectively [10, 11].

Genes related to phenotypes of interest: GeneCards is a database for searching comprehensive information regarding human genes [12]. We searched the GeneCards database for genes associated with SARS-CoV-2 and cardiomyocyte injury, using the keywords "SARS-CoV-2" and "cardiomyocyte injury". Molecular Signatures Database (MSigDB) provides extensive gene sets for gene set enrichment analysis [13]. According to the significant pathophysiological processes of SARS-CoV-2 infection in hiPSC-CMs obtained by subsequent analyses, we retrieved related genes from the MsigDB database with phenotypes of interest as keywords.

### Differential expression analysis and principal component analysis (PCA)

A differential expression analysis was performed on GSE184715 and GSE150392 by using the DESeq2 package (version 1.40.2) in R software. The differentially expressed genes (DEGs) were selected with the criteria |logFC|>1, adj. *p*<0.05. Our analysis involved intersecting the DEGs with genes related to "SARS-CoV-2" and "cardiomyocyte injury", and displaying these genes with Venn diagrams. A heatmap of DEGs was drawn using pheatmap package (version 1.0.12). PCA analysis and visualization were performed on two datasets using the prcomp and plot functions in R software.

### Protein-protein interaction (PPI) analysis

STRING is a database able to retrieve direct or indirect interactions between proteins [14]. We constructed a protein-protein interaction network using the STRING database (www.string-db.org) to perform PPI analysis with a minimum interaction score of 0.400, and Cytoscape (version 3.9.1) software [15] was applied for network visualization. MCODE plugin was used to cluster the PPI networks, and the clusters were recombined according to their biological functions for subsequent analysis. Top 10 genes were screened as hub genes with the MCC method of cytoHubba plugin.

### Functional enrichment analysis

On the WebGestalt website [16], we conducted gene ontology (GO) and pathway analysis, combining biological process (BP), cellular component (CC), molecular function (MF), and Kyoto Encyclopedia of Genes and Genomes (KEGG), Reactome, WikiPathways databases. Significant GO and pathway terms with *p*<0.05 were visualized via ggpubr package (version 0.6.0) and ggplot2 package (version 3.4.2).

### Hub gene analysis

GSE184715 and the validation datasets were used to validate the expression of the top hub genes and perform ROC analysis, visualized by the ggplot2 package. To perform functional similarity analysis, we calculated the semantic similarity of GO terms applying the GOSemSim package (version 2.26.0) [17]. With the corrplot package (version 0.92), we analyzed the correlation between the top 10 hub genes.

### Upstream transcription factor (TF) prediction

The NetworkAnalyst web-based platform provides a comprehensive analysis of gene expression profiles [18]. TF binding profiles are available in JASPAR, a public database for searching and predicting transcription factors (TFs) [19]. The upstream TFs of a gene were predicted using the JASPAR database on NetworkAnalyst. Our analysis intersected the predicted upstream TFs of the top 10 hub genes of the four groups that were clustered and recombined to obtain their common upstream TFs. Analysis of common upstream TFs was conducted using GO, pathway, and PPI methods. Using the Coremine Medical database, we predicted drugs that would target the common upstream TFs.

### Drug prediction

Coremine Medical (https://www.coremine.com/medical/) is an online tool to search, update, and share medical information, and it was used to predict drugs for the top 5 hub genes of the four groups that were clustered and recombined. By intersecting the drug prediction results, we obtained the final predicted drugs based on biological significance.

### Expression visualization

Based on the differential expression analysis of clustered genes, top 10 genes, and genes related to phenotypes of interest in GSE184715, we used the ggpubr package and ggplot2 package to visualize the results. Cytoscape was used to visualize predicted TF and drug network results.

### Statistical analysis

In order to analyze and plot the data, R software (version 4.3.0) was used, and SPSS software (version 26.0) was used to analyze the statistical data. ROC analysis was used to validate the diagnostic performance of top 10 genes, comparison between continuous variables was performed with t-test or Wilcoxon rank sum test as needed, and two-sided *p*-value <0.05 was considered statistically significant.

## Result

**Fig. 1:**
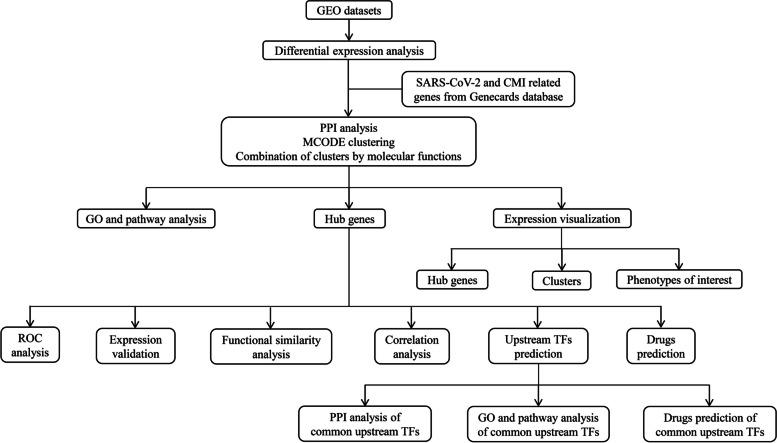
Flow chart of this study. CMI: cardiomyocyte injury; GEO: Gene Expression Omnibus; GO: gene ontology; PPI: protein-protein interaction; ROC: receiver operating characteristic; SARS-CoV-2: severe acute respiratory syndrome coronavirus 2; TF: transcription factor

In this study, we attempted to explore the potential biological mechanisms underlying the development of SARS-CoV-2 infection with hiPSC-CMs by employing comprehensive network-based transcriptome analysis methods, including functional enrichment analysis, PPI analysis, hub gene analysis, upstream TF prediction and drug prediction with the aim of revealing a series of pathophysiological processes including mitochondrial dysfunction (Fig. 1). In this study, the aim is to investigate the physiological mechanisms underlying SARS-CoV-2 infection in hiPSC-CMs, reveal pathophysiological processes, including mitochondrial dysfunction, and investigate potential intervention targets and therapeutic agents (Fig. 1).

### Differential expression analysis and PPI analysis

**Fig. 2:**
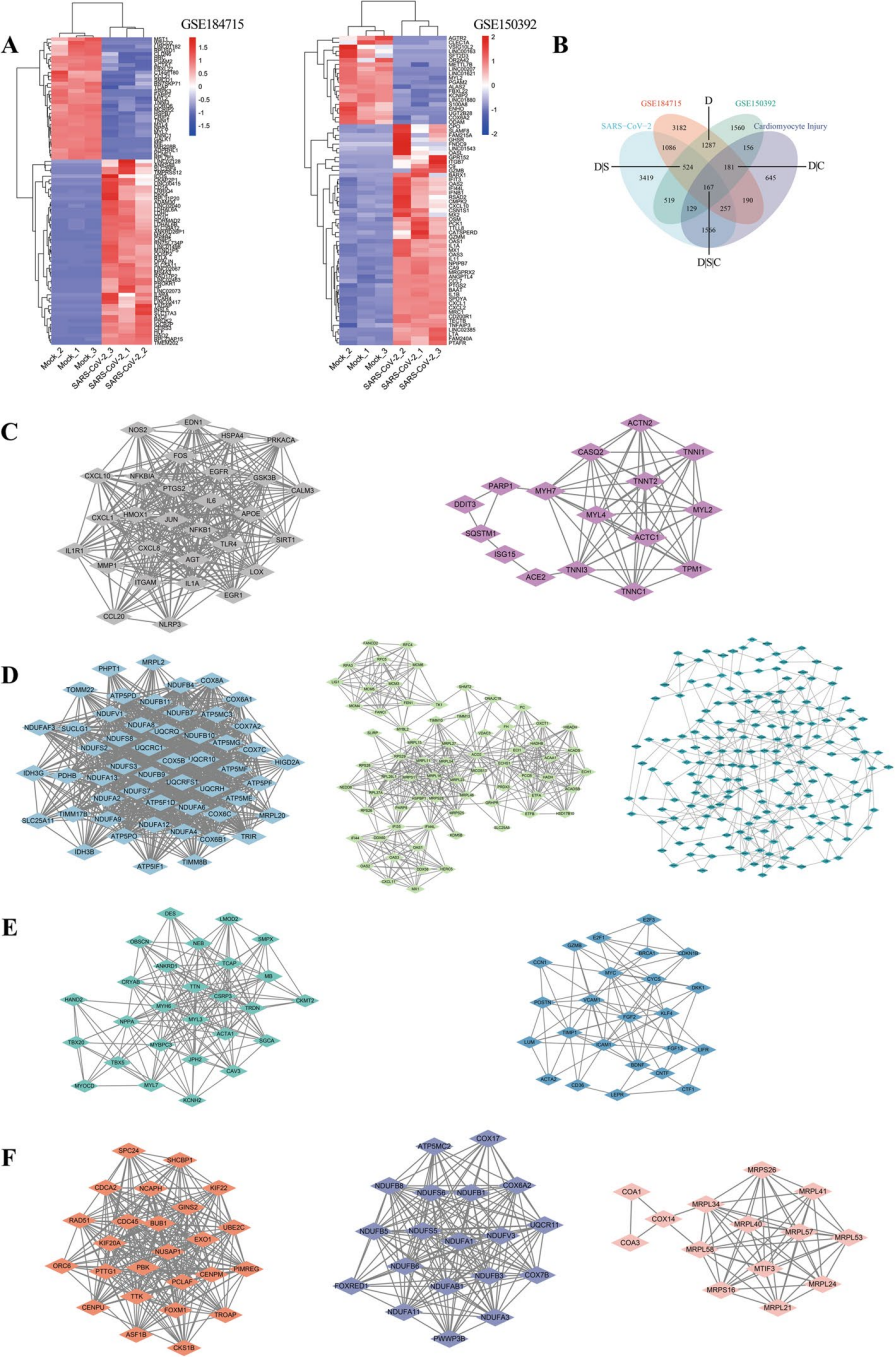
Differential expression analysis and PPI network construction clustered by MCODE plugin of Cytoscape software. **A** Heatmap of DEGs of GSE184715 and GSE150392. **B** Venn diagram of DEGs of GSE184715 and GSE150392, as well as SARS-CoV-2 and CMI related genes from GeneCards database. **C** PPI network of genes of D|S|C cluster 1 and D|S|C cluster 2. **D** PPI network of genes of D|S cluster 1, D|S cluster 2 and D|S cluster 3. **E** PPI network of genes of D|C cluster 1 and D|C cluster 2. **F** PPI network of genes of D cluster 1, D cluster 2 and D cluster 3. DEGs: differently expressed genes; C: CMI; D: common DEGs of GSE184715 and GSE150392 excluding genes associated with SARS-CoV-2 and CMI; S: SARS-CoV-2; other abbreviations as in Fig. 1

GSE184715 and GSE150392 were selected for analysis and DEGs were obtained. PCA analysis revealed significant distinctions in the principal component space between samples from the SARS-CoV-2 infected group and the mock group across both two datasets (Supplementary Figure 1). We screened DEGs from GSE184715 and GSE150392 using the criteria |logFC|>1, adj. *p *< 0.05. A total of 6874 DEGs were found in GSE184715, while 4523 DEGs were found in GSE150392 (Fig. 2A). The number of DEGs is too large for direct hub gene analysis and PPI analysis. In order to find genes related to SARS-CoV-2 and cardiomyocyte injury, we searched GeneCards database with key words "SARS-CoV-2" and "cardiomyocyte injury". Based on the intersection of these two datasets and the two gene lists, “DEGs AND SARS-CoV-2 AND CMI (D|S|C)”, “DEGs AND SARS-CoV-2 NOT CMI (D|S)”, “DEGs NOT SARS-CoV-2 AND CMI (D|C)”, and “DEGs NOT SARS-CoV-2 NOT CMI (D)” were obtained (Fig. 2B). We tracked the four gene sets with PPI analysis, using MCODE to cluster them based on their biological significance (Fig. 2C-F). The parameters set by MCODE were as follows: D|S|C: Degree Cutoff: 6, Node Score Cutoff: 0.2, Max. Depth from Seed: 100; D|S: Degree Cutoff: 16, Node Score Cutoff: 0.6, Max. Depth from Seed: 100: D|C: Degree Cutoff: 6, Node Score Cutoff: 0.4, Max. Depth from Seed: 100; D: Degree Cutoff: 6, Node Score Cutoff: 0.2, Max. Depth from Seed: 100. The clustered genes were then recombined based on their functional similarity. In the end, we were able to isolate four distinct groups, i.e., Group 1, Group 2, Group 3 and Group 4, which indicated the presence of cardiomyocyte cytoskeletal protein, proto-oncogenes and inflammation, mitochondria, and cytoplasm, respectively.

### Integrated analysis of four groups with distinct biological functions

**Fig. 3:**
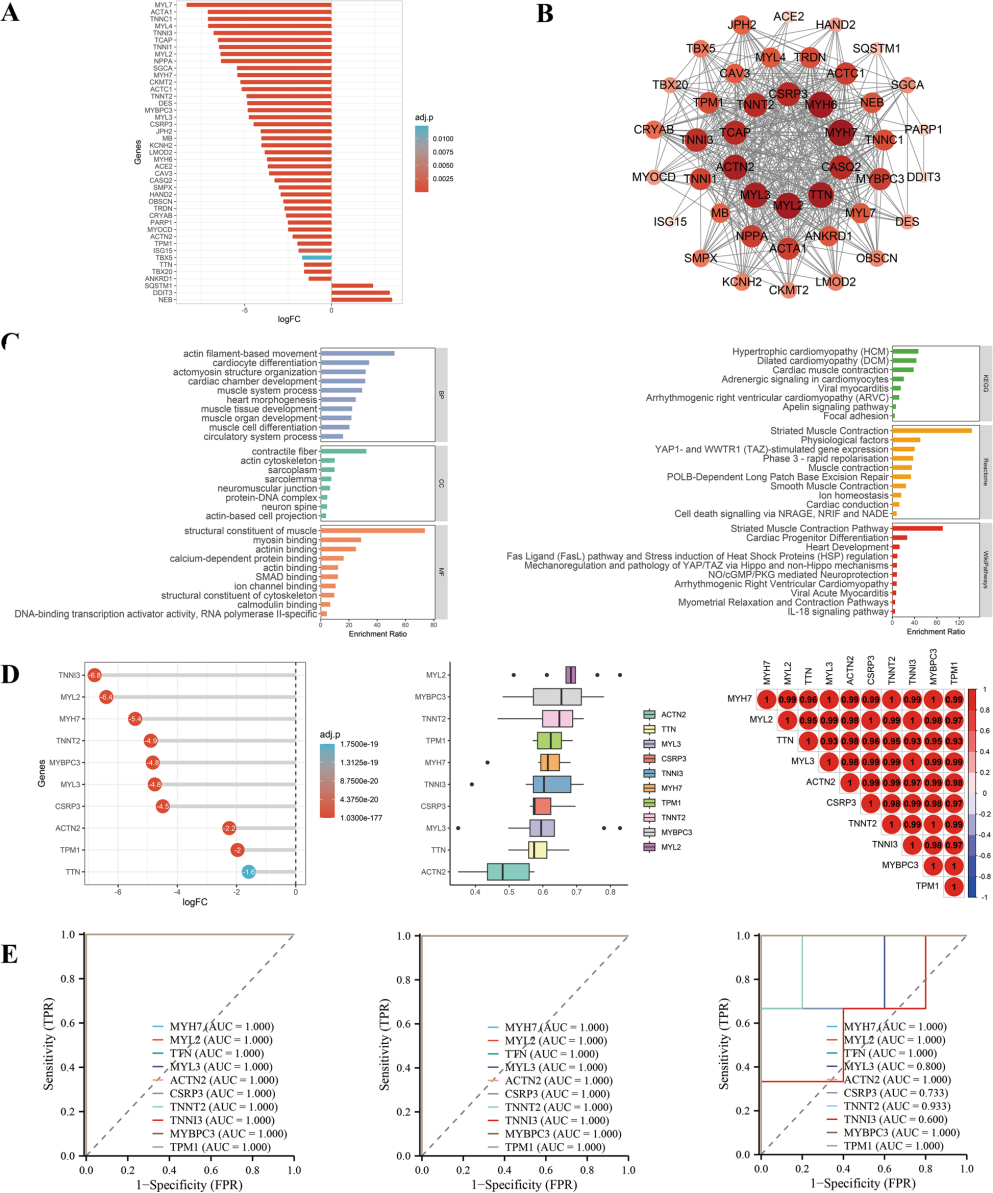
Integrated analysis of Group 1. **A** Expression visualization of Group 1 genes. **B** PPI network of Group 1 genes, and the size and color of nodes represent the degree values calculated by Cytoscape software. **C** GO and pathway analysis of Group 1 genes. **D** Expression visualization, functional similarity analysis and correlation analysis of top 10 hub genes among Group 1, and top 10 hub genes were calculated by the MCC method in cytoHubba plugin of Cytoscape software. **E** ROC curves of top 10 hub genes among Group 1 in GSE184715, GSE193722 and GSE169241. BP: biological process; CC: cellular component; KEGG: Kyoto Encyclopedia of Genes and Genomes; MF: molecular function; Group 1: combination of D|S|C cluster 2 and D|C cluster 1; other abbreviations as in Figs. 1, 2

From Fig. 3, there was a significant downregulation of expression in most of the genes in Group1. Downregulated genes were mostly related to cardiomyocyte cytoskeletal proteins, such as myosin, actin, tropomyosin, ACE2. There was an enrichment of genes within Group1 involved in myocardial contraction, actin filament movement, actin filament bundle formation, myofibril structure, and other pathways associated with myocardial contraction, and these gene functions were downregulated in Group1. According to pathway analysis methods, Group1 is associated with myocardial contractility, myocardial disease. Furthermore, all 10 hub genes showed a significant downregulation of expression, good functional similarity and positive correlations. As shown by ROC curves, all top 10 genes were qualified for diagnostic prediction in GSE169241, indicating good accuracy. In GSE193722 and GSE169241, the top 10 genes were validated well.

**Fig. 4:**
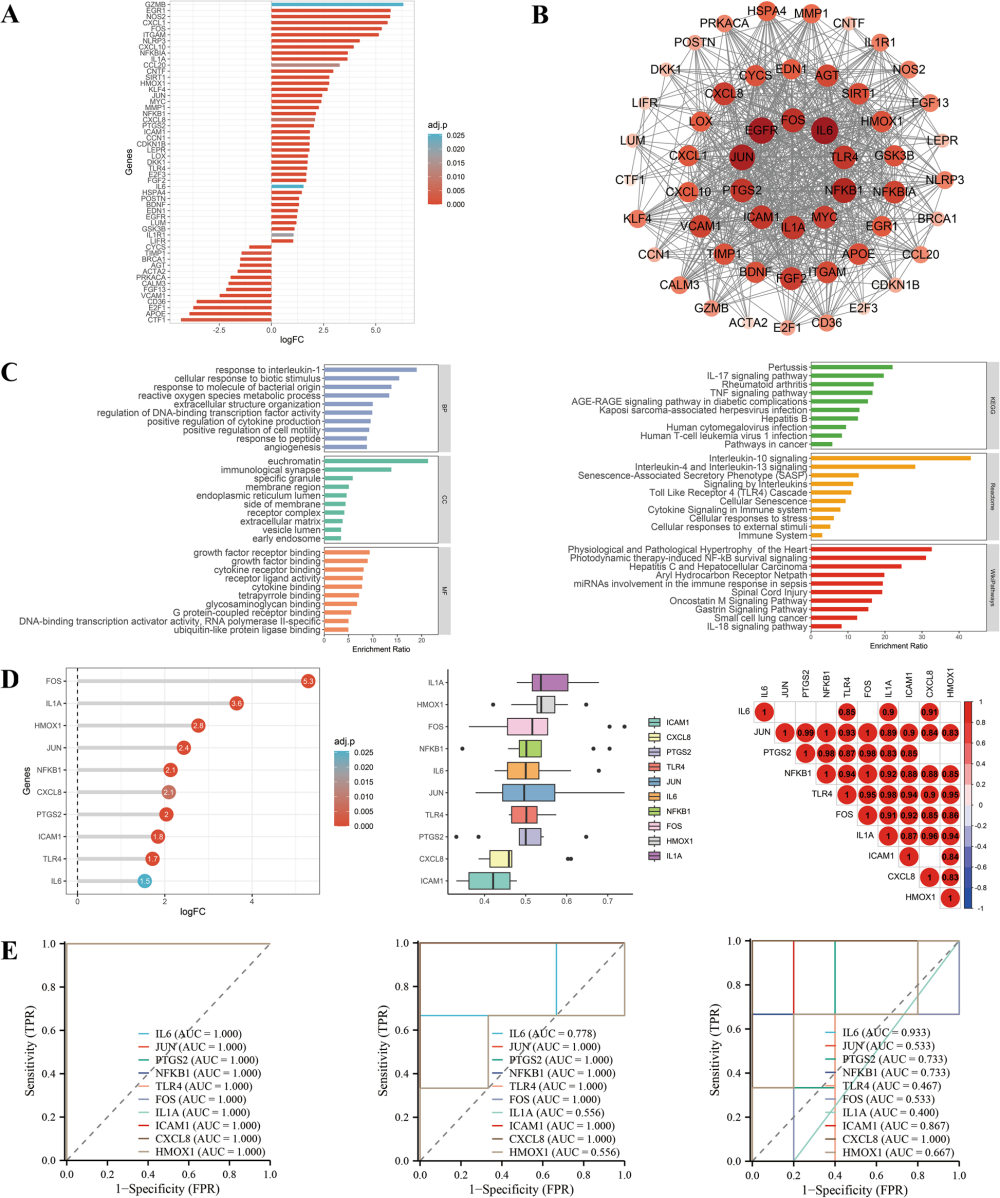
Integrated analysis of Group 2. **A** Expression visualization of Group 2 genes. **B** PPI network of Group 2 genes, and the size and color of nodes represent the degree values calculated by Cytoscape software. **C** GO and pathway analysis of Group 2 genes. **D** Expression visualization, functional similarity analysis and correlation analysis of top 10 hub genes among Group 2, and top 10 hub genes were calculated by the MCC method in cytoHubba plugin of Cytoscape software. **E** ROC curves of top 10 hub genes among Group 2 in GSE184715, GSE193722 and GSE169241. Group 2: combination of D|S|C cluster 1 and D|C cluster 2; other abbreviations as in Figs. 1, 2 and 3

We can see from Fig. 4, there were major downregulations in Group2, including antioxidants, ion channel proteins, while upregulations included proto-oncogenes, inflammation genes and nuclear factor-kappaB (NF-κB) pathway-related genes. Response to inflammation or infections, cytokine production was enriched in genes in Group2. Additionally, pathway analysis revealed that these genes tended to be enriched in biological processes such as tumor necrosis factor (TNF) pathway, Toll like receptor 4 (TLR4) pathway, NF-κB pathway. Proto-oncogenes upregulated in this study did not necessarily pertain to cell division. Instead, these genes increased cellular transcription function to protect cells as well as activate pro-inflammatory pathway genes. Top 10 hub genes were classified into proto-oncogenes and inflammation that were upregulated. These genes exhibited overall favourable functional similarity, while a good correlation was found when correlation was calculated between them. An effective diagnostic predictive performance was demonstrated by the ROC curves of the top 10 genes in GSE169241, and the two cell datasets illustrated a strong diagnostic predictive capability in cells. Top 10 genes had unsatisfactory validation results in the GSE169241 dataset and good validation results in GSE193722. The GSE169241 dataset, derived from mixed tissue, may explain the suboptimal validation due to the presence of various cell types beyond cardiomyocytes.

**Fig. 5:**
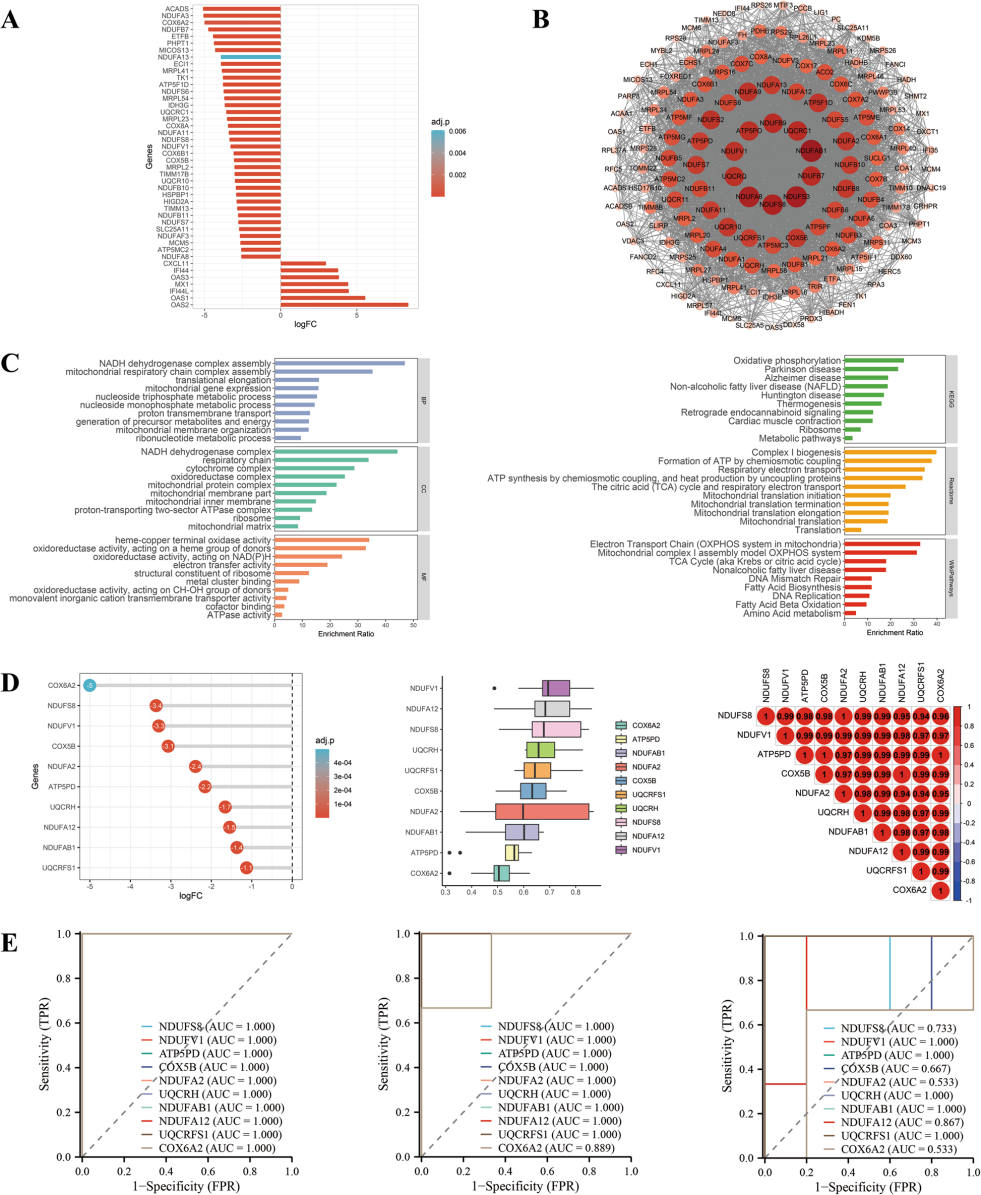
Integrated analysis of Group 3. **A** Expression visualization of Group 3 genes. **B** PPI network of Group 3 genes, and the size and color of nodes represent the degree values calculated by Cytoscape software. **C** GO and pathway analysis of Group 3 genes. **D** Expression visualization, functional similarity analysis and correlation analysis of top 10 hub genes among Group 3, and top 10 hub genes were calculated by the MCC method in cytoHubba plugin of Cytoscape software. **E** ROC curves of top 10 hub genes among Group 3 in GSE184715, GSE193722 and GSE169241. Group 3: combination of D|S cluster 1, D|S cluster 2, D cluster 2 and D cluster 3; other abbreviations as in Figs. 1, 2 and 3

We can obtain from Fig. 5, there was clear evidence of mitochondrial dysfunction in this part of the genes that were downregulated. Nicotinamide adenine dinucleotide (NADH) dehydrogenase, mitochondrial respiratory chain (MRC), electron transfer dysfunction, and adenosine triphosphate (ATP) synthesis malfunction were enriched in genes belonging to Group3. According to pathway analysis, these genes were enriched for genes involved in ATP synthesis, electron transfer abnormalities, and oxidative phosphorylation. In the MRC, three gene families have been significantly downregulated, including ATP synthase, NADH:ubiquinone oxidoreductase family (NDUFs), and cytochrome c oxidase (COX). A significant disruption of mitochondrial biological functions was also observed when hiPSC-CMs were infected with SARS-CoV-2. The top 10 hub genes were downregulated, functionally similar and positively correlated. The ROC curves of the top 10 genes in the two cell datasets indicated accurate diagnostic prediction, and the results in GSE169241 similarly pointed to accuracy. The validation of the top 10 genes was conducted well on the GSE193722 and GSE169241 datasets.

**Fig. 6:**
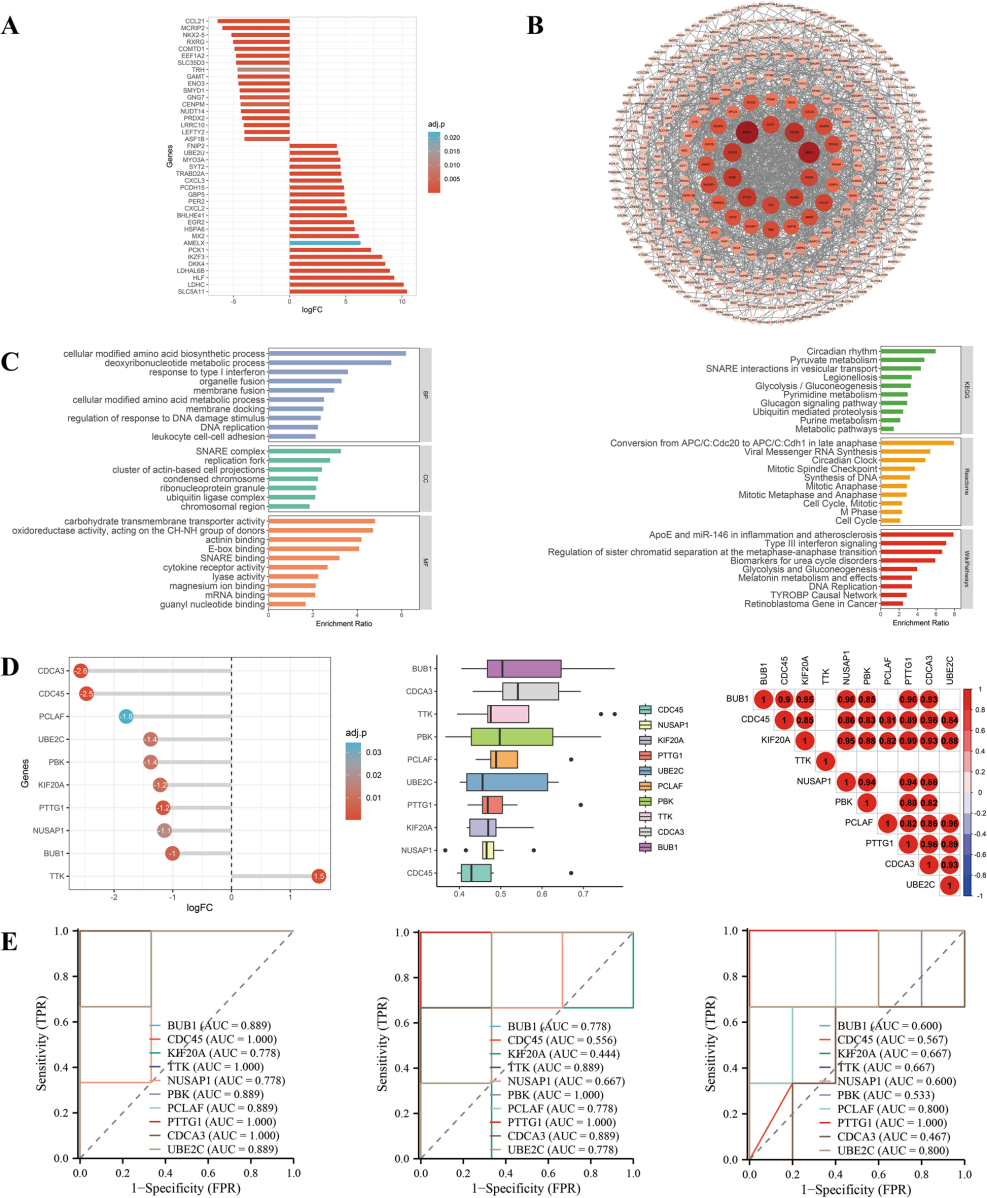
Integrated analysis of Group 4. **A** Expression visualization of Group 4 genes. **B** PPI network of Group 4 genes, and the size and color of nodes represent the degree values calculated by Cytoscape software. **C** GO and pathway analysis of Group 4 genes. **D** Expression visualization, functional similarity analysis and correlation analysis of top 10 hub genes among Group 4, and top 10 hub genes were calculated by the MCC method in cytoHubba plugin of Cytoscape software. **E** ROC curves of top 10 hub genes among Group 4 in GSE184715, GSE193722 and GSE169241. Group 4: combination of D|S cluster 3 and clusters of D other than cluster 2 and cluster 3; other abbreviations as in Figs. 1, 2 and 3

From Fig. 6, cell metabolism, cell division and inflammation-related genes constituted the majority of hub genes in Group4. Biological processes such as glucose metabolism, amino acid metabolism, fatty acid metabolism, pyruvate metabolism and citric acid cycle, and other important substance synthesis and degradation were found to be enriched in Group4 genes. The cell localization revealed that most of these processes took place in intracellular transport and in the cytoplasm. Thus, most biologic processes that occur in the cytoplasm are affected. Top 10 hub genes were downregulated except TTK, meaning cell replication, functionally similar and positively correlated. The robust diagnostic predictive ability was illustrated by ROC analysis of the top 10 genes in both GSE169241 and the two cell datasets. In the GSE169241 tissue-sourced dataset, hub genes validation was poor; while in the GSE193722 cell-sourced dataset, validation was similarly less satisfactory. In addition to the possible influence of cellular and tissue factors on validation, the heterogeneity of expression of these genes in different cells and tissues may also be a factor.

### Expression visualization of significant biological functions

MsigDB was searched for corresponding gene lists with several keywords, and gene expression was calculated and visualized for GSE184715. From Supplementary Figure 2, the analysis showed upregulation of (A) the NF-κB pathway, (B) proto-oncogenes, and (H) interferon signaling genes, indicating that these functions were also differentially regulated. In contrast, (C) cardiac cytoskeletal protein, (D) ATP synthesis and electron transport chain (ETC), (E) replication, (F) transcription, (G) translation, etc., had downregulated gene functions.

In GSE184715, we calculated and visualized the expression of genes in corresponding gene lists with several keywords in MsigDB. In Supplementary Figure 3, we found that (A) glucose metabolism, amino acid metabolism, fatty acid metabolism, pyruvate metabolism and citric acid cycle, nucleic acid metabolism, (B) ubiquitination, (C) autophagy, and (D) endocytosis, phagocytosis, exocytic vesicle, cytoplasmic vesicle related genes had downregulated biological functions.

### Prediction of upstream TFs and drugs of hub genes

**Fig. 7:**
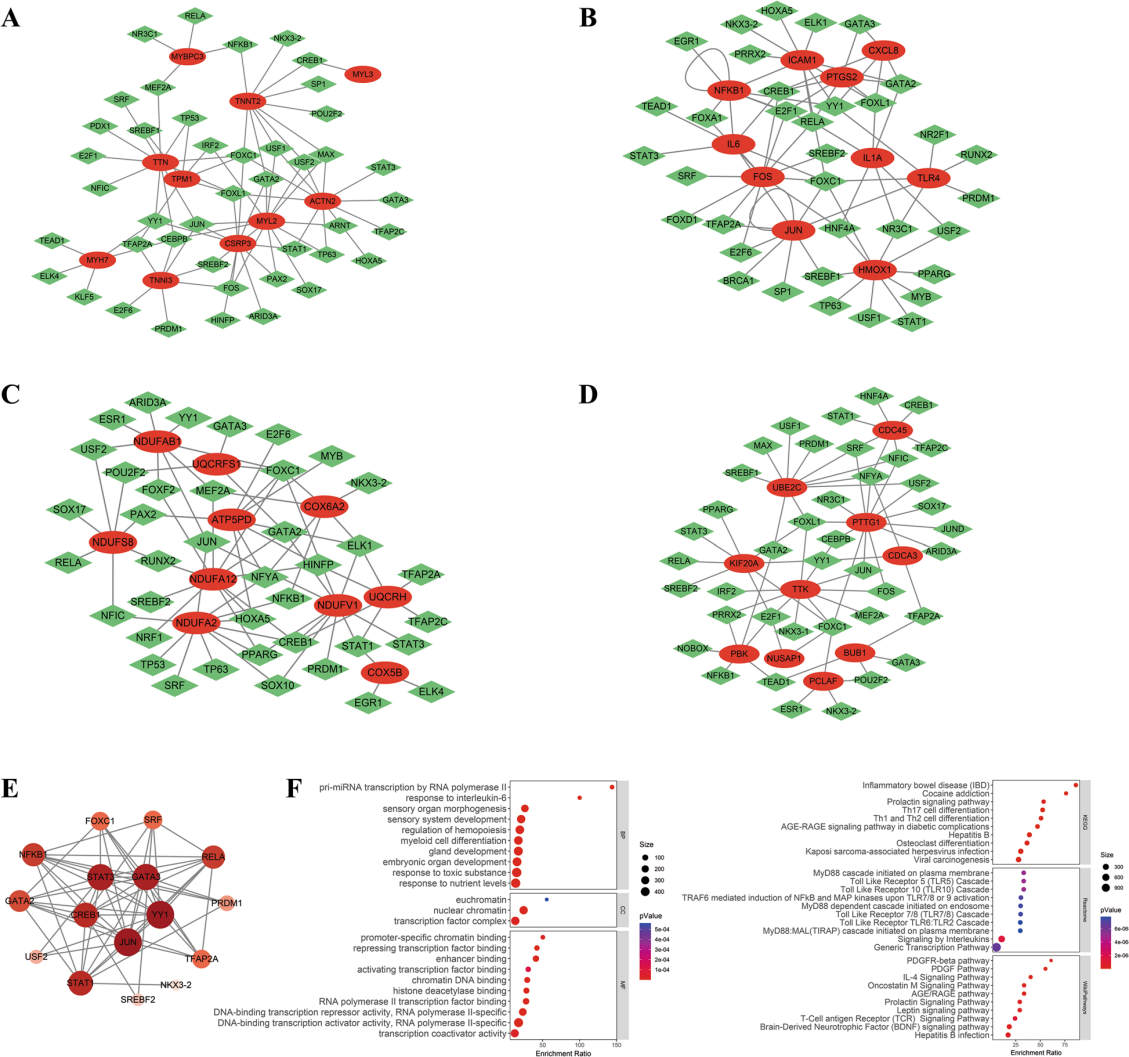
TF-gene regulatory network construction by JASPAR database in NetworkAnalyst online tool. The regulatory network between upstream TFs and top 10 hub genes among **A** Group 1, **B** Group 2, **C** Group 3, and **D** Group 4. **E** PPI network of common upstream TFs of top 10 hub genes among 4 groups, the size and color of nodes represent the degree values calculated by Cytoscape software, and top 5 TFs in the center were calculated by the MCC method in cytoHubba plugin of Cytoscape software. **F** GO and pathway analysis of common upstream TFs. Abbreviations as in Figs. 1, 2 and 3

We can see from Fig. 7, the hub genes of the four groups were predicted to have upstream TFs, which were intersected to determine 16 TFs. These TFs were ranked by hub gene scores and the most important five TFs were JUN, YY1, GATA3, CEBPB and STAT3. There was an enrichment of gene transcription and inflammation activation in the biological functions and molecular properties of these 16 TFs. Inflammation-related pathways were identified by pathway analysis for these TFs.

**Fig. 8:**
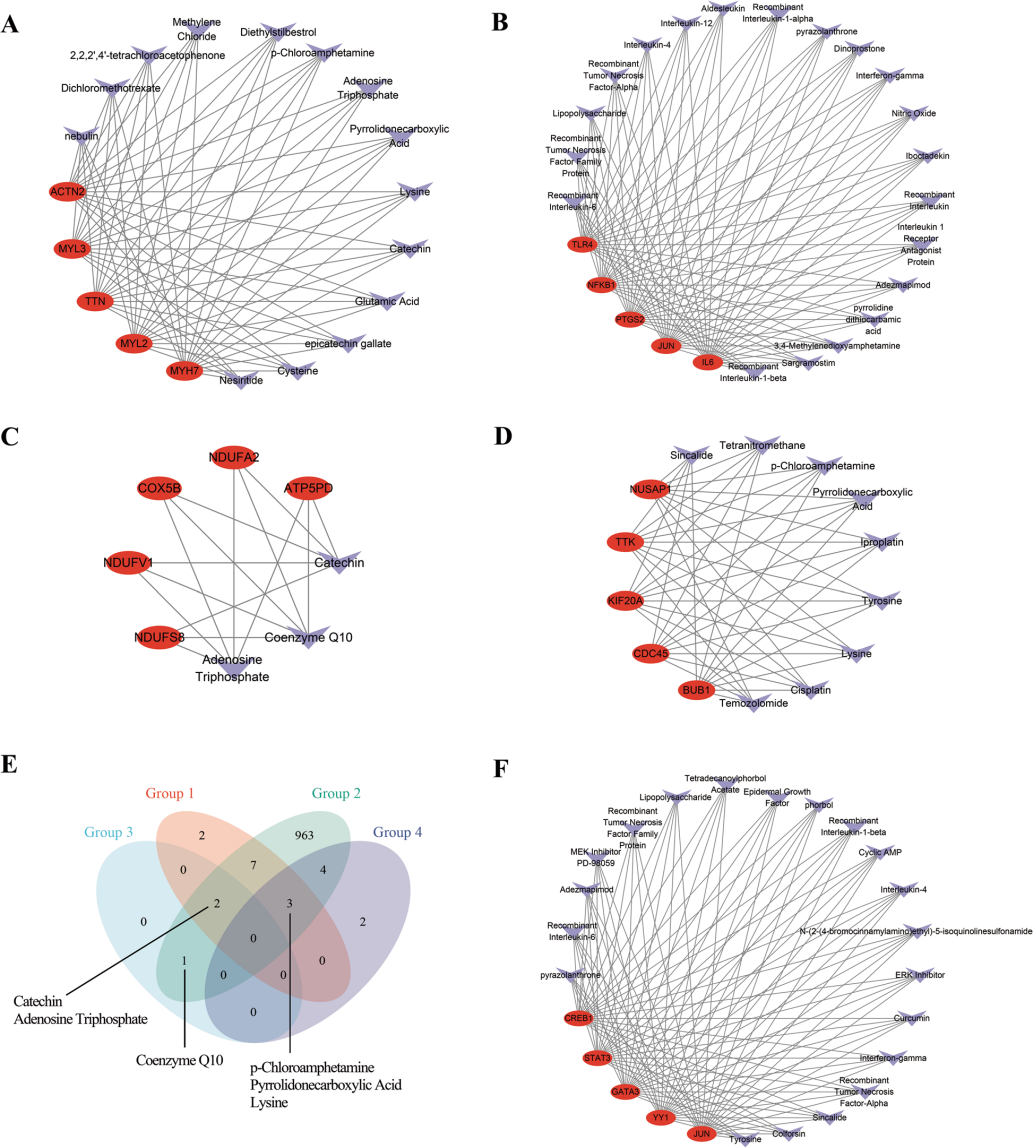
Identification of candidate drugs by Coremine Medical database. Predicted drugs of top 5 hub genes among **A** Group 1, **B** Group 2, **C** Group 3, and **D** Group 4. **E** Venn diagram of predicted drugs of top 5 hub genes among 4 groups. **F** Predicted drugs of top 5 common upstream TFs of top 10 hub genes among 4 groups. Abbreviations as in Figs. 1, 2

Based on the hub genes of the four groups, we predicted drugs and intersected the predicted drugs. From Fig. 8, there are several drugs in this class, including coenzyme Q10, catechin, adenosine triphosphate, lysine, p-chloroamphetamine, pyrrolidonecarboxylic acid.

Coenzyme Q10 is essential for the ETC in mitochondrial aerobic respiration and is used to protect against viral myocarditis. Catechins, present in green tea, serve as antioxidants. Adenosine triphosphate (ATP) fuels energy metabolism across sugars, fats, and proteins. Lysine, an essential amino acid, promotes pyruvate metabolism, influencing respiratory chain processes. However, the pharmacological mechanisms of pyrrolidonecarboxylic acid and p-Chloroamphetamine remain unknown.

**Fig. 9:**
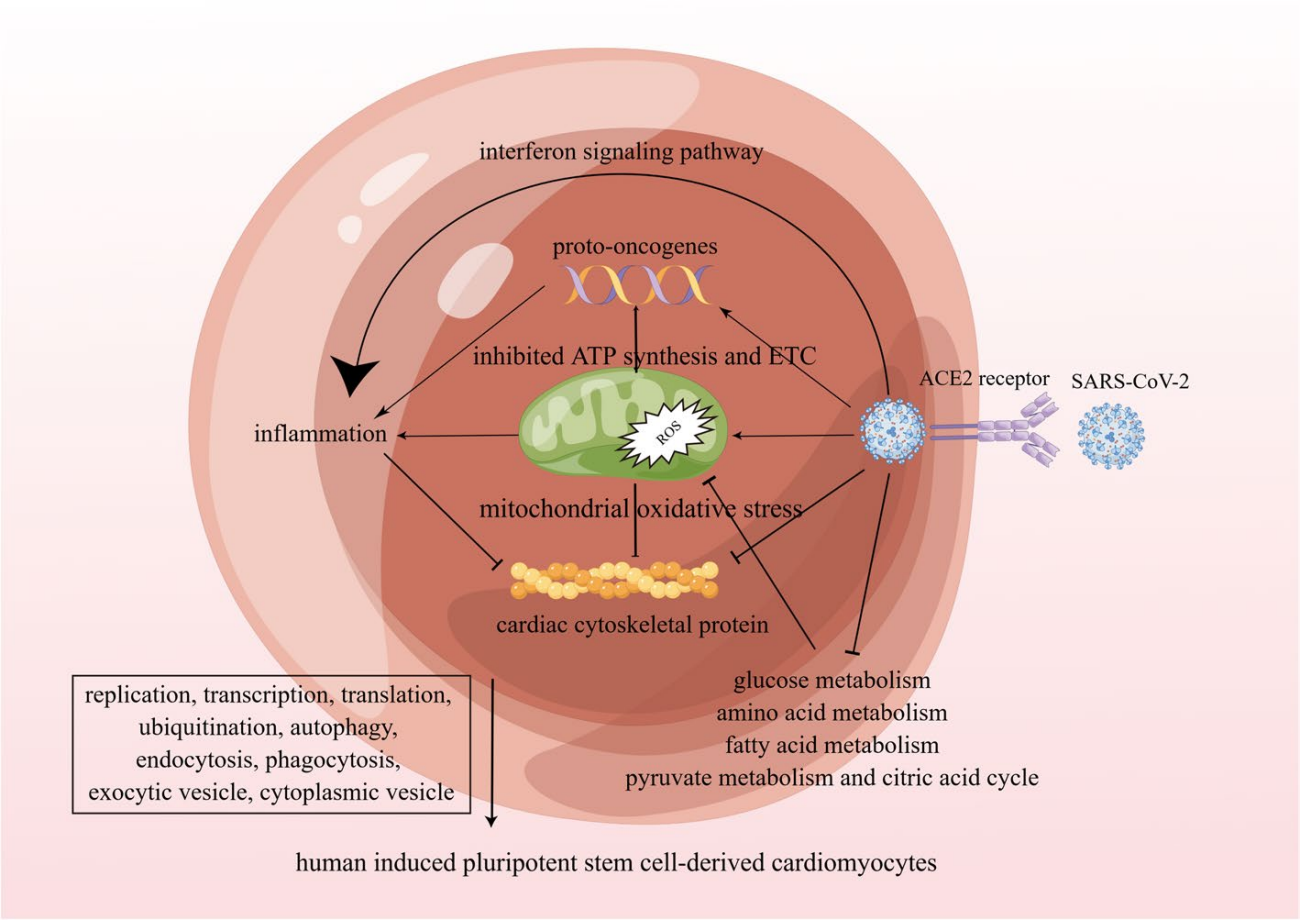
Pathophysiological process of SARS-CoV-2 infecting hiPSC-CMs. ACE2: angiotensin-converting enzyme 2; ATP: adenosine triphosphate; ETC: electron transport chain; hiPSC-CMs: human induced pluripotent stem cell-derived cardiomyocytes; ROS: reactive oxygen species; SARS-CoV-2: severe acute respiratory syndrome coronavirus 2

Figure 9 illustrates the process of infection with SARS-CoV-2 in hiPSC-CMs. A number of major findings were drawn from the previous analyses, including downregulation of cardiomyocyte cytoskeletal proteins, mitochondrial dysfunction, activation of proto-oncogenes, and downregulation of cellular life processes dependent on energy metabolism. The ACE2 receptor is a conduit for the invasion of hiPSC-CMs by SARS-CoV-2. The virus activated the interferon pathway and proto-oncogenes, triggered mitochondrial oxidative stress, and directly inhibited four major biochemical processes within cardiomyocytes. Virus infection impairs the synthesis and metabolism of basic intracellular substances and reduces the raw materials for mitochondrial energy generation. Furthermore, SARS-CoV-2 infection activates proto-oncogenes, and mitochondrial oxidative stress results in mitochondrial dysfunction and energy abnormalities, ultimately causing the body to lack energy. Inflammatory responses are triggered by activation of the interferon pathway, upregulation of proto-oncogenes, and mitochondrial oxidative stress. Cardiomyocyte contraction is impacted by these factors, which leads to heart failure. The cell's physiological functions are impacted by factors such as replication, transcription, translation, ubiquitination, autophagy, and cellular transport. Thus, mitochondrial dysfunction plays a central role in the entire pathophysiology of SARS-CoV-2-infected hiPSC-CMs.

## Discussion

It is possible to simulate SARS-CoV-2 infection using hiPSC-CMs to study the myocardial cell damage caused by COVID-19 [8, 20]. At the transcriptome level, this study explores the following questions. First, it is imperative to understand what biological processes occur in SARS-CoV-2-infected hiPSC-CMs. In addition, the second important scientific question is how these functions are regulated. Biological processes are the third key scientific question, so what are the key interventions and strategies? In order to answer these questions, our study performed transcriptome analysis. We were happy to find that our results were as anticipated.

Our findings confirm those from previous reports that hiPSC-CMs infected with SARS-CoV-2 showed reduced myocardial contraction, mitochondrial redox dysfunction, interferon pathway activation, and inflammation pathway activation and calcium imbalance [21–23]. Infected hiPSC-CMs were found to have extensive intracellular myogenic fibre fragmentation due to SARS-CoV-2 infection (Perez-Bermejo et al., 2021) [21]. Alternatively, Tangos et al. (2022) observed COVID-19 autopsy cardiac studies that showed pro-inflammatory hyperresponsiveness, increased oxidative stress, and impaired cardiomyocyte function [22]. Further, Zhang et al. (2023) suggested that SARS-CoV-2 infection of hiPSC-CMs can trigger metabolic dysregulation and decreased contractile function [23]. These studies, however, have been limited by the experimental conditions in which they were conducted, which prevented them from resolving the underlying causes of myofibre damage, oxidative stress, and abnormal metabolism in cardiomyocytes. However, our findings go beyond that. After splitting and combining DEGs and GeneCards database according to protein function, we further analyzed the results of PPI. The study confirmed previous findings in bioinformatics, as well as discovered new ones. Aside from the pathophysiological changes mentioned above, SARS-CoV-2 infection also inhibited the important biological process in hiPSC-CMs: the three major metabolisms, replication, transcription, translation functions, cell communication and substance transport and other normal biological functions. It has been shown that SARS-CoV-2 infection of hiPSC-CMs caused cytoskeleton protein dysfunction, mitochondrial dysfunction, upregulation of proto-oncogenes and activation of the inflammation pathway in cardiomyocytes, but the intermediate regulatory process is not clear. Based on transcriptome analysis and relevant references, we draw the following inferences.

Cells with mitochondria are more susceptible to SARS-CoV-2 infection than other types, according to a study [24]. Cardiomyocytes and skeletal muscle cells have high mitochondrial content so they are more susceptible to infection [25]. There is evidence that mitochondria reduce ETC complex I and ATP synthase expression, increase apoptosis-inducing genes, and aggravate hypoxia in epithelial cells [26]. It has been found that three MRC gene families, including ATP synthase, NDUFs and COX, were significantly downregulated during our study. An increased production of reactive oxygen species (ROS) was induced by the mitochondrial ETC and inflammation, while an inhibited ATP synthase led to an ATP deficiency [27]. COVID-19 patients' heart failure is clinically linked to decreased myocardial contractility, which translates to an abnormal contraction of cardiomyocyte protein cytoskeletons. Various causes can lead to cardiomyocyte cytoskeleton protein dysfunction. ROS damage, ATP deficiency, and inflammation are likely to contribute to cardiomyocyte cytoskeleton protein dysfunction in the model of SARS-CoV-2 infection of hiPSC-CMs [28]. In the presence of increased ROS, energy disorders, inflammation, and cardiomyocyte cytoskeleton protein dysfunction, antioxidant genes such as CAT and SOD2 were significantly downregulated. In response to downregulation of the antioxidant genes SOD2 and CAT, activator protein-1 (AP-1) DNA binding activity and mRNA levels for c-fos and c-jun were enhanced [29]. We found that antioxidant genes, including CAT and SOD2, were significantly downregulated, suggesting that antioxidants had been depleted. JAK/STAT signaling pathway can be activated by interferon, which upregulates many transcription factors, promotes inflammatory factors, and reduces myosin heavy chain, myosin light chain and actin expression [30]. Proto-oncogenes were activated by cyclic adenosine monophosphate (cAMP) generation [31]. A GO analysis of its cellular localization revealed that it occurred in mitochondria. This study revealed the fundamental role of mitochondrial dysfunction in the infection of hiPSC-CMs with SARS-CoV-2.

It has been shown that mitochondrial dysfunction plays a fundamental role in the pathophysiology of hiPSC-CMs infected with SARS-CoV-2. The mitochondria produce more than 95% of the ATP produced by the heart. An inadequate amount of ATP is produced when fatty acid metabolism, glucose metabolism, and mitochondrial function are impaired [32]. Normal cellular biological activities, including gene transcription, DNA replication, and protein translation, as well as many aspects of protein modification, such as ubiquitination and acetylation, and cellular and mitochondrial autophagic processes, are affected [33, 34]. Biological processes such as these are dependent upon the mitochondria's functionality and are critical to maintaining cell health. There was a downregulation of replication, transcription, and translation genes, particularly RNA polymerase and ribosome-related genes. According to these results, transcription and translation were impaired in the host cell, genetic information flow from nucleic acids to proteins was inhibited, and intercellular communication was disrupted [35, 36]. Absence of basic cellular metabolites and important biological processes are supported by downregulation of genes involved in glucose metabolism, fatty acid metabolism, protein metabolism, and nucleic acid metabolism. The tricarboxylic acid cycle is the final metabolic pathway of glucose metabolism, fatty acid metabolism, protein metabolism, and also the hub of glucose, fat, amino acid metabolism, providing reducing equivalents for oxidative phosphorylation reaction to generate ATP. The metabolic processes involving glucose, fat, amino acids, and tricarboxylic acid were inhibited, resulting in a further reduction in ATP synthesis. There may be potential therapeutic opportunities for hiPSC-CMs infected with SARS-CoV-2 by targeting metabolic disorders [37].

It is also important to note that other important biological functions were inhibited, including ubiquitination and intracellular substance transport. Ubiquitination can modify target proteins and participate in the regulation of alm metabolism. It is an active energy-consuming biological behavior. Researchers have confirmed that SARS-CoV-2 hijacked the ubiquitination process in cells, causing an upregulation of the expression of ubiquitin-related genes in immune cells and alveolar cells [38]. Our analysis revealed a decrease in ubiquitination-related gene expression, likely caused by mitochondrial dysfunction and abnormal ATP synthesis. These biological functions also require energy and are active energy-consuming processes. They include endocytosis, phagocytosis, and extracellular vesicle function. With the decrease of ATP synthesis, these cell functions were also inhibited [39].

Our findings suggest that mitochondrial dysfunction lies at the center of the infection of hiPSC-CMs with SARS-CoV-2. The following are potential approaches that may treat COVID-19 heart damage: 1. Blocking the entry of SARS-CoV-2 from ACE2 receptors into the cell is the fundamental solution; 2. Regulation of fatty acid and glucose metabolism; 3. Supplementation with antioxidants to alleviate the mitochondrial oxidative stress; 4. Inhibition of intracellular inflammation; and 5. Protection of cardiac fibres or cardiac strengthening therapy. From these directions, we believe potential therapeutic approaches can be developed for treating COVID-19 cardiac damage.

Our hub gene validations were confirmed at the cellular and tissue levels using multiple datasets, increasing their credibility. As a result of this comprehensive validation strategy, the findings of the study can be taken into account in a variety of biological levels, which enhances their reproducibility. Nonetheless, there must be an appreciation of organism variability and the differences in experimental conditions, which may result in differing validation results. Moreover, different laboratory designs and conditions may also have an impact on the validation results.

The study dealt well with the three major scientific questions. Our innovations and highlights are listed below. As part of our methodological approach, we classified DEGs into four groups based on biological functions, and then analyzed them. We also explored other cell functions that had not been described previously. The results of the transcriptome analysis also allowed us to examine the regulatory mechanisms of many biological functions and signaling pathways. Several clinically effective drugs were also suggested as possible intervention targets.

Due to the limited sample size of the dataset, machine learning algorithms were not used to identify predictive gene signatures or biomarkers between infected and non-infected hiPSC-CMs. Even though machine learning could be valuable, we preferred to emphasize the validity and rationale of alternative analysis methods (e.g., the MCC method of the cytoHubba plugin to obtain hub genes). A network perturbation analysis and pathway crosstalk analysis tools were not used in the study. We made this decision based on the technical complexity of these methods and the belief that the current team may be unable to adequately cope with these methods' requirements. While network perturbation analysis has the advantage of revealing systematic insights into biological networks and predicting system responses, pathway crosstalk analysis tools are uniquely suited to reveal interactions within pathways and 'crosstalk' between different pathways, providing valuable insights into the full understanding of biological system behavior [40, 41]. For a thorough explanation of our findings, we used other methods to probe deeper into the relationship between the affected pathways.

Proteomics and metabolomics, which are multi-omics technologies, will help provide a comprehensive view of intracellular molecular activity in the future. As a result of these integrated approaches, we can gain a deeper insight into the underlying biological mechanisms that link gene expression, protein levels, and metabolic activities. To verify the credibility of bioinformatics analysis results in living organisms, we simulate more complex and realistic physiological environments with animal models. By using clinical samples of cardiac tissues associated with SARS-CoV-2 infection to integrate laboratory findings with clinical outcomes, we can improve our understanding of pathophysiological changes and guide future therapeutic interventions by analyzing patient samples.

Our bioinformatics analysis revealed plausible insights into the biological functions and regulatory mechanisms of signaling pathways. Yet, a deeper understanding of the true targets and regulatory pathways necessitates further investigation at cellular and animal levels. Identified drugs show clinical promise, with some proven safe, while others require additional scrutiny. For less-explored drugs, ongoing cellular and animal studies are essential to elucidate their mechanisms of action. Additional treatments for mitochondrial dysfunction could be explored.

## Conclusions

As a result of infection of hiPSC-CMs with SARS-CoV-2, several pathophysiological processes are triggered, including disorders of material metabolism, damage to myocardial backbone proteins, mitochondrial oxidative stress, inflammatory responses, and other energy-related processes. Of particular interest is the fact that mitochondrial dysfunction appears to be particularly critical in this array of pathophysiological processes. Thus, therapies that target mitochondrial dysfunction may be an effective method of alleviating COVID-19's cardiac complications. Study findings provide a foundation for further analysis and understanding of the cardiac pathophysiological processes triggered by SARS-CoV-2 infection, as well as for future drug development strategies.

### Supplementary Information

**Supplementary Material 1.**

**Supplementary Material 2.**

**Supplementary Material 3.**

## Data Availability

The data used to analyze in this study could be found in the GEO database: https://www.ncbi.nlm.nih.gov/geo/query/acc.cgi?acc=GSE184715; https://www.ncbi.nlm.nih.gov/geo/query/acc.cgi?acc=GSE150392; https://www.ncbi.nlm.nih.gov/geo/query/acc.cgi?acc=GSE193722; https://www.ncbi.nlm.nih.gov/geo/query/acc.cgi?acc=GSE169241. The original contributions presented in the study and the code are included in the article and supplementary material, further questions and reasonable request for the code can be directed to the corresponding author.
