# Supplementary figures and images for: Decoding HiPSC-CM’s Response to SARS-CoV-2: mapping the molecular landscape of cardiac injury
Source: BMC Genomics. 2024 Mar 12;25:271. doi: 10.1186/s12864-024-10194-5 (PMC10936015; doi:10.1186/s12864-024-10194-5)

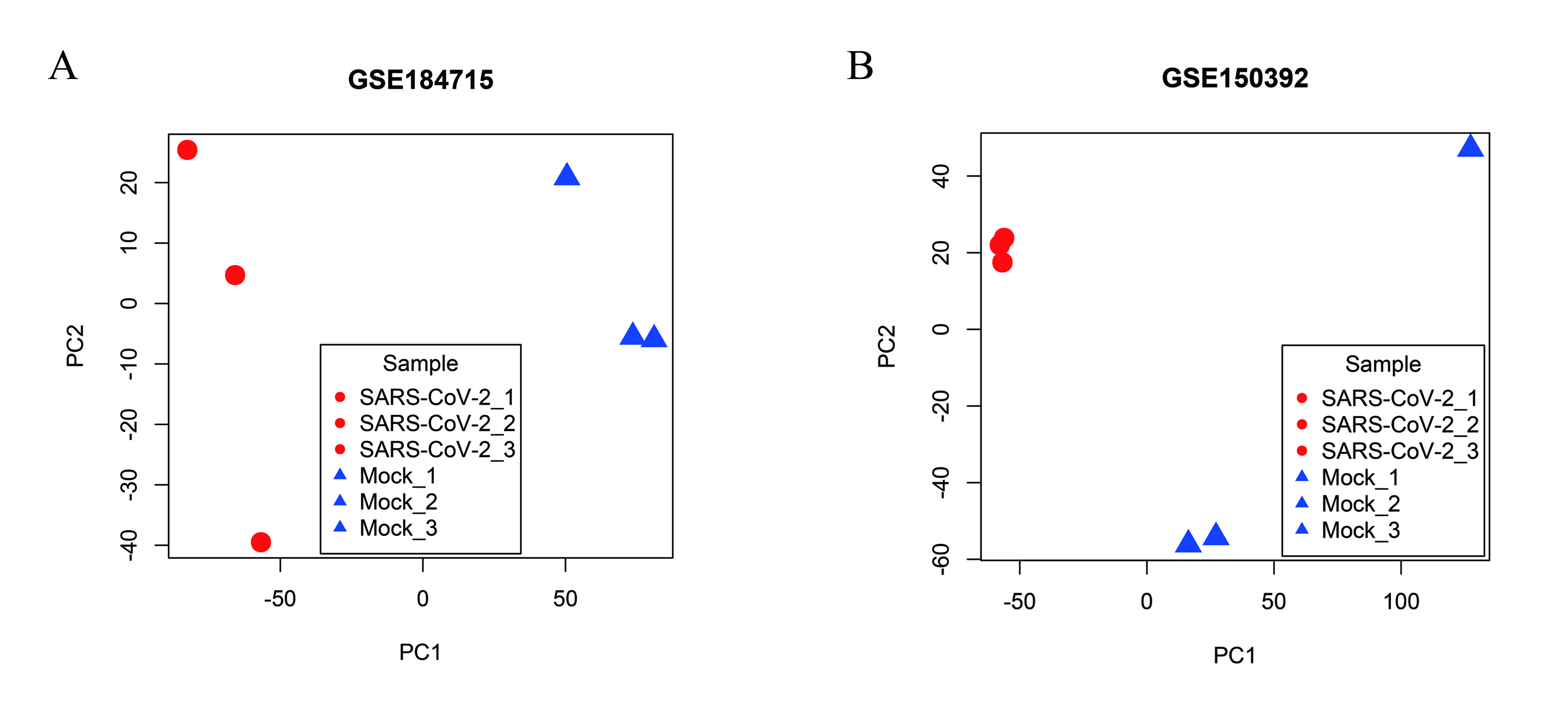

Supplement: Supplementary file 1 — Supplementary Material 1. [file 12864_2024_10194_MOESM1_ESM.tif]
